# The O_2_, pH and Ca^2+^ Microenvironment of Benthic Foraminifera in a High CO_2_ World

**DOI:** 10.1371/journal.pone.0050010

**Published:** 2012-11-15

**Authors:** Martin S. Glas, Katharina E. Fabricius, Dirk de Beer, Sven Uthicke

**Affiliations:** 1 Biogeochemistry Department, Microsensor Group, Max Planck Institute for Marine Microbiology, Bremen, Germany; 2 Australian Institute of Marine Science, Townsville, Queensland, Australia; Argonne National Laboratory, United States of America

## Abstract

Ocean acidification (OA) can have adverse effects on marine calcifiers. Yet, phototrophic marine calcifiers elevate their external oxygen and pH microenvironment in daylight, through the uptake of dissolved inorganic carbon (DIC) by photosynthesis. We studied to which extent pH elevation within their microenvironments in daylight can counteract ambient seawater pH reductions, i.e. OA conditions. We measured the O_2_ and pH microenvironment of four photosymbiotic and two symbiont-free benthic tropical foraminiferal species at three different OA treatments (∼432, 1141 and 2151 µatm *pCO_2_*). The O_2_ concentration difference between the seawater and the test surface (ΔO_2_) was taken as a measure for the photosynthetic rate. Our results showed that O_2_ and pH levels were significantly higher on photosymbiotic foraminiferal surfaces in light than in dark conditions, and than on surfaces of symbiont-free foraminifera. Rates of photosynthesis at saturated light conditions did not change significantly between OA treatments (except in individuals that exhibited symbiont loss, i.e. bleaching, at elevated *pCO_2_*). The pH at the cell surface decreased during incubations at elevated *pCO_2_*, also during light incubations. Photosynthesis increased the surface pH but this increase was insufficient to compensate for ambient seawater pH decreases. We thus conclude that photosynthesis does only partly protect symbiont bearing foraminifera against OA.

## Introduction

Ocean acidification has become a major threat to our world’s oceans [1]. From preindustrial times until today, atmospheric carbon dioxide (*pCO_2_*) concentrations increased from ∼280 ppm to >390 ppm, and are predicated to rise to ∼800 ppm by the end of this century under the IPCC business-as-usual emission scenario (WG 1, A2, [2]), which is likely to be exceeded [1], [3]. The current rapid atmospheric CO_2_ increase is mostly due to anthropogenic induced changes from increased fossil fuel combustion, deforestation and changes in land use and is now greater than at any time in the last 300 million years of Earth’s history [4], [5], [6]. Not only is CO_2_ a potent greenhouse gas in the atmosphere resulting in global warming, but about one third of the anthropogenic CO_2_ increase is taken up by the oceans [1], [7]. This uptake reduces pH and consequent carbonate saturation state (Ω) of the ocean surface waters, a process generally termed as ‘ocean acidification’ (OA). Phototrophic marine calcifiers (such as coccolithophores, foraminifera, calcareous algae and corals) strongly contribute to the cycling of carbon in our world’s oceans, as part of the so called ‘biological pumps’ [8]–[10]. By changes in ocean chemistry ocean acidification poses a direct threat to most calcifying organisms and consequently the biological pumps [1], [11], [12].

However, the effect of bulk seawater pH is mediated through the diffusive boundary layer (DBL), which governs transport resistance between the bulk seawater and the organisms’ surface. Around phototrophic organisms (including most major calcifiers such as phytoplankton, foraminifera, corals and calcareous algae) DBLs can maintain substantial gradients of O_2_ and pH to the bulk seawater, due to their high photosynthetic and respiratory activity [13]–[21]. Especially under daylight conditions, surface pH levels of phototrophic or photosymbiotic organisms can differ strongly (>0.1 pH units) from the surrounding seawater [13]–[21]. It is this surface pH and the resulting gradients within the organisms’ DBL, rather than the bulk seawater pH, which determine ion-availability [17] and consequently transport kinetics between the tissues and surrounding seawater. Microenvironmental pH dynamics are therefore likely to play an important role in physiological responses to ocean acidification. Understanding O_2_ and pH dynamics and variability within the DBLs under both present day and future OA conditions is therefore essential for all transport involving metabolic processes such as calcification, photosynthesis or respiration.

We hypothesize that OA induced increases of seawater DIC might enhance photosynthesis of photosymbiotic calcifiers and consequently result in increased pH levels on their surfaces in daylight. Thus, the pH DBL might form a shield around the organism protecting it from OA. We studied whether this pH elevation within their microenvironment can protect photosymbiotic calcifiers (or at least partly compensate) from the effects of ocean acidification in daylight and therefore lend additional resistance compared to non photosymbiotic calcifiers. We tested this hypothesis by measuring the O_2_ and pH microenvironment of 4 photosymbiotic and 2 symbiont-free benthic tropical foraminiferal species under different ocean acidification scenarios in light and dark conditions.

Benthic foraminifera represent a good group of model organisms for this study, because compared to most other calcifiers, calcification is periodic rather than continous, and periods of calcification can be detected visually. Additionally, the process of chamber formation is very sensitive to mechanical disturbances and thus unlikely to occur in short term flume measurements (see material and methods section, also [22]–[23], reviewed in [24]). Impacts of active calcification on pH microenvironments can thus be excluded during the measurements. In addition, both symbiont-free and photosymbiotic species were tested, allowing for the direct comparison of the effects of net photosynthesis and respiration on O_2_ and pH microenvironments under equal experimental conditions.

## Materials and Methods

### Sampling and Culturing

Specimens of the photosymbiotic species *Marginopora vertebralis*, *Amphistegina radiata, Heterostegina depressa*, and *Peneroplis* sp., and the symbiont-free species *Quinquelloculina* sp. and *Miliola* sp. were hand collected from coral rubble and other substrates containing foraminiferal assemblages by SCUBA diving during a cruise in the summer months of 2010 in the Whitsunday area, central section of the Great Barrier Marine Park. All necessary permits were obtained prior to field collection from the Great Barrier Marine Park Authority (Permit-No: G09/30237.1). Collection sites included, Bait Reef S 19°80.17′ E 149°07.55′, Daydream Island S 20°15.35′, E 148°48.73′, Shaw Island S 20°31.02′ E 149°04.48′ and Deloraine Island S 20°09.30′, E 149°04.50′ (depth 5–13 m, seawater temperature during collection 28.8±0.2°C (mean ± SD) and salinity 35–36). A detailed description of the sampling sites can be found in Uthicke et al. [25].

After collection, specimens were washed off substrates, cleaned by gentle washing and sieving and identified to species and genus level [26] under a dissecting-microscope (Leica MX16 A, Solms, Germany). Samples were kept in natural seawater (24° - 26°C) under low light conditions (10 µmol photons m**^−^** ^2^ s**^−^** ^1^), until they were transported to the Australian Institute of Marine Science (AIMS) in Townsville. Prior to experiments, specimens acclimatised in indoor climatic chambers>3 weeks in natural seawater (replaced every 3 days, sediments removed) at 24° - 26°C, 10 µmol photons m**^−^** ^2^ s**^−^** ^1^, 12 h : 12 h diurnal cycling and fed with microalgae (*Isocrysis* sp.). Salinity of nearshore seawater available at the AIMS was diluted (32–34) due to high seasonal rainfall. During culturing and experimental treatments seawater salinity was therefore adjusted to 35 by the addition of sea salt (Sunray, Cheetham Salt, Melbourne, Australia). Salinities were measured using a refractometer (S/Mill-E, Atago, Tokyo, Japan).

### Experimental Setup

Carbon perturbations experiments were performed by the addition of CO_2_ enriched air into a semi-closed circulation system of filtered (1 µm) natural seawater. CO_2_ enriched air (0.2%) was humidified via a system of Erlenmeyer flasks and bubbled into an aerated reservoir tank (30 L), connected to incubation chambers, which contained the organisms (water flow rate 0.5–1.0 cm s^−1^). Gas flow rates and thereby *pCO_2_* levels were regulated via mass flow controllers (accuracy 1.5%, GFC17, Aalborg, Orangeburg, NY, USA). The system was allowed to equilibrate for>48 h.

All amperometric and potentiometric microsensor measurements were conducted in a Faraday cage to minimize electrical disturbance. Before the measurements specimens were carefully transferred with a fine brush from the incubation chambers into a flow-cell (1.2 ml volume), connected to the same circulation system. Net flow rates within the flow cell were adjusted volumetrically to 0.50±0.02 cm s**^−^** ^1^ (mean ± SD), to simulate average natural *in situ* flow conditions experienced by epifaunal and shallow infaunal foraminifera within the benthic boundary layer of reef environments [27]. Net horizontal flow was monitored ∼3 mm above the foraminiferal surface by observing particle movements via a stereo-microscope (K400, Motic, Xiamen, China).

Illumination was provided from above via a fiber-optic guide from a halogen light source (Schott KL2500, Mainz, Germany). Light intensities were monitored with a quantum irradiance meter (LI-250A, LI-COR, Lincoln, NE, USA), combined with a light sensor for photosynthetic active radiation (PAR).

### Microelectrodes

Clark-type O_2_ microsensors with a guard cathode (tip diameter ∼20 µm,<1 s response time (t_90_), precision 0.05 µM) were constructed and calibrated as previously described [28]. pH measurements were performed by liquid ion exchange (LIX) membrane microelectrodes (tip diameter 5–20 µm,<1 s response time (t_90_), precision 0.001, on the NBS scale), as previously described by de Beer [29], and a commercial pH meter (pH 1100, Oakton, Vernon Hills, IL, USA). Ca^2+^ concentrations were determined with LIX microelectrodes (tip diameter 5–20 µm,<2 s response time (t_90_), precision 13 µM), which were prepared, calibrated and used as described [30], [31]. A detailed description of the measurement setup can be found in Polerecky *et al*. [32].

### Experimental Procedure and Determination of Microenvironmental Dynamics

**Figure 1:**
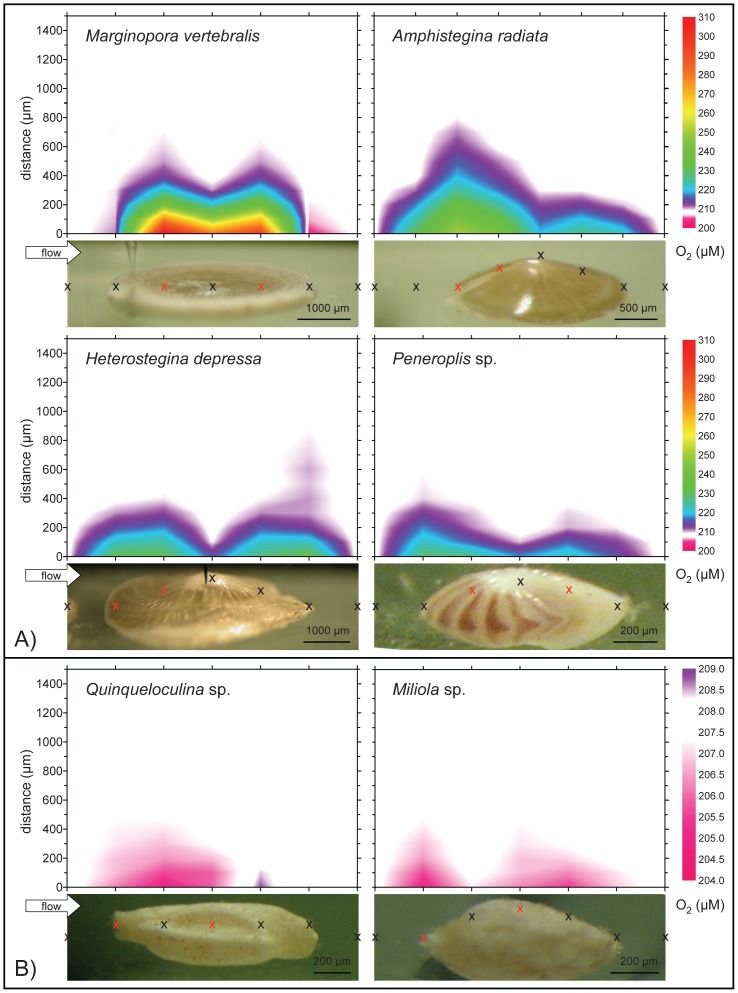
Microenvironmental O_2_ heterogeneity at a *pCO_2_* of 432 µatm, 30 µmol photons m^−2^ s^−1^ light, and 0.5 cm s^−1^ water flow across foraminiferal shell surfaces. Data derived from fine-scale microsensor profiles at the points indicated by the crosses. Red crosses indicate the measurement positions (n = 2–4) used for the calculation of means per individual. Note the different contour scales between A) photosymbiotic and B) symbiont-free species.

Using a fine brush, foraminifera were positioned horizontally in the middle of the flow cell resting on their central elevations, with the exception of *Marginopora vertebralis,* which exhibits a flat surface structure (Figure 1). Microsensor tips were positioned on the calcite shell surfaces of foraminifera, using a stereo-microscope and a 3D-manual micromanipulator (MM33, Maerzhaeuser, Wetzlar, Germany). O_2_ evolution within the DBL of phototrophic species was tested under varying light intensities (data not shown). A light intensity of 30 µmol photons m**^−^** ^2^ s**^−^** ^1^ was found saturating for all photosymbiotic species, without causing photo-inhibition in the tested low light species *Amphistegina radiata* and *Heterostegina depressa* [27] and used throughout all ‘light’ experiments (see also [33], [34]).

To determine the t_90_ value of steady-state signals of the system O_2,_ pH and Ca^2+^ probes were positioned on the test surface of photosymbiotic individuals and recorded for ∼30 min, while light levels were altered (light/dark changes). O_2_ (pA) reached>90% steady-state signals ∼2 min, pH values (mV) took<6 min, while Ca^2+^ (mV) values did not change significantly. To ensure steady-state, light levels were applied for 10–60 min prior to measurements. Steady-state profiles were measured in step sizes of 50 µm (up to 400 µm) and 100 µm about 1500 µm upward perpendicular to the foraminiferal test, through the diffusive boundary layer into the bulk seawater (Figure 1). Due to slow erecting of individuals by rhizopodial movements, gentle nudges with a fine brush were applied in between profiles to assure rhizopodial retraction, so that foraminifera and their extending DBLs remained in their horizontal position.

To illustrate the effect of zero flow (i.e. static culture) conditions on pH microenvironments, individuals of *Marginopora vertebralis* were pH profiled at the same position on the calcite shell at 432 µatm (pH 8.22), 30 µmol photons m**^−^** ^2^ s**^−^** ^1^ under mean turbulent flow conditions (0.5 cm s**^−^** ^1^) and consecutively after flow was turned off after 5, 10, 20, 30, 40, 50, 60, 90 and 100 min and again after flow was re-established within 5 min. Individuals of *M. vertebralis* were chosen for these measurements since they remained attached to the bottom of the flow cell in a fixed position for extended periods of time.

To estimate spatial microenvironmental O_2_ heterogeneity across the shell surfaces, specimens of every species were fine scale profiled at 432 µatm from front to back in flow direction (Figure 1).

### Determination of O_2_, H^+^ and Ca^2+^ Microenvironmental Dynamics

To account to some extent for spatial heterogeneity across shell surfaces (Figure 1) during the profiling experiment, foraminiferal specimens (n = 2) were profiled in 2–4 locations on their calcite shell during the experiment (indicated by red crosses in Figure 1). To determine possible treatment effects on O_2_ dynamics and to evaluate exact placement of microsensor tips for consecutive measurements, individuals were profiled with O_2_ microsensor at 432 µatm in light, prior to each treatment incubation. Profiling experiments were conducted at a *pCO_2_* of 432 µatm (pH of 8.22; ambient), 1142 µatm (pH 7.85) and 2151 µatm (pH 7.60) with photosymbiotic species, and at two *pCO_2_* levels (432 and 2151 µatm) with symbiont-free individuals (Table 1). After 24 h of incubation, microsensor measurements across the DBL of all specimens in both light (30 µmol photons m**^−^** ^2^ s**^−^** ^1^) and darkness were conducted for O_2_ on day 2, pH on day 3, and Ca^2+^ on day 4.

**Table 1:** Experimental parameters (mean±SD), monitored each day of each 4-day *pCO_2_* incubations (n = 4) beside TA, which was sampled at the end of each experiment (n = 1).

	Measured Input Parameters						Calculated (n = 4)						
	T [°C](n = 4)	TP [µmolkg^−1^](n = 4)	TSi [µmolkg^−1^](n = 4)	pH (NBS)(n = 4)	DIC [µmolkg^−1^](n = 4)	TA [µmolkg^−1^](n = 1)	TA[µmol kg^−1^]	*pCO_2_*[µatm]	CO_2(aq)_[µmol kg^−1^]	HCO_3_^–^[µmol kg^−1^]	CO_3_^2−^[µmol kg^−1^]	Ω_Ca_	Revelle factor
*pCO_2_* treatment													
432 µatm	25.9±0.2	0.08±0.01	27.19±0.28	8.22±0.01	2343±43	2710	2709±43	432±17	12.0±0.5	2060±41	271±5	6.52±0.12	9.54±0.12
1141 µatm	26.0±0.4	0.04±0.01	23.31±0.58	7.85±0.01	2468±21	2622	2617±23	1141±28	31.5±0.8	2307±20	129±3	3.11±0.08	14.12±0.16
2151 µatm	25.9±0.4	0.09±0.01	23.51±0.86	7.60±0.03	2603±36	2651	2654±28	2151±179	59.2±4.5	2465±35	78.7±3.7	1.90±0.09	16.81±0.17

Abbreviations: TP = total phosphorus, TSi = total silicate, DIC = dissolved inorganic carbon, TA = total alkalinity, *pCO_2_* = partial pressure of CO_2_, Ω_Ca_ = saturation state of calcite, Revelle factor = (ΔCO2_(aq)_/CO2_(aq)_)/(ΔDIC/DIC).

All measured input parameters, beside TA, were used for CO_2_SYS calculations.

### Monitoring of Treatments

Seawater was renewed for each experimental treatment and kept at a constant salinity (35) and pH according to the treatment (Table 1). Temperature, pH, DIC, total silicate and total phosphorus were monitored daily. DIC samples were filtered (0.2 µm nylon filters), stored gas tight, head-space free at 4°C and analysed within a week by flow injection analysis [35]. Samples for nutrient analyses (including total silicate and phosphorus) were filtered (0.2 µm nylon filters), immediately frozen and consequently analysed with a Bran and Luebbe AA3 segmented flow analyzer (Norderstedt, Germany) following Ryle et al. [36]. Samples for total alkalinity (TA) were taken at the end of each experiment, filtered (0.2 µm nylon filters), poisoned with HgCl_2_ and kept at 4°C until being shipped to the University of Sydney, where they were analysed by open cell potentiometric titration [37], and calculated using linear Gran plots [38]. Corrections were applied based on certified reference material (A. Dickson, Scripps Institution of Oceanography, CA, USA).

### Assessment of Individuals

For microsensor measurements, healthy, intact foraminiferal specimens of similar size and pigment shading were selected and liveliness confirmed in all individuals by the observation of movement. Individuals were photographed (Canon 30D, Tokio, Japan) via the dissecting microscope, before and after the experimental treatments (for complete sets, see Figure S1, S2, S3). At the end of the experiments, individuals were examined and photographed under a fluorescence microscope (Axioskop mot plus, Carl Zeiss, Goettingen, Germany) equipped with a digital camera (AxioCamMRc5, Carl Zeiss, Goettingen, Germany). Fluorescence images were obtained using a halogen lamp for incident light and DAPI (excitation, G365 nm; dichroic mirror FT395; emission LP420 nm) and FITC (excitation, BP 450–490 nm; dichroic mirror FT510; emission LP515 nm) filter sets (Carl Zeiss, Goettingen, Germany). Foraminiferal sizes (longest diameter) were measured in small individuals from microscopic images by the software AxioVision (version 4.8.1, Carl Zeiss, Goettingen, Germany) and in large individuals via a digital calliper.

### Carbonate Chemistry Calculations

Calculations based on measurements of DIC, pH, temperature, salinity, total-phosphate and silicate (Table 1) were performed in CO_2_SYS [39], using K1 and K2 according to Millero et al. [40], with dissociations constants for H_2_SO_4_ detailed in Dickson [41]. Measured and calculated levels of total alkalinity deviated <0.2%, indicating that carbonate chemistries were in equilibrium throughout the experiments (Table 1).

### Data and Statistical Analysis

Hydrogen ion (H^+^) concentrations for dilute aqueous solutions were calculated from pH levels. Differences in concentrations between the bulk seawater and the surface of the shells, denoted as ΔO_2_, ΔH^+^ and ΔCa^2+^, were attained from the measured profiles. Concentration differences were calculated as the lowest and highest spatial points of the profiles respectively. At very low metabolic rates and therefore increased resolution, profile noise was balanced by a line of best fit through the seawater baseline concentrations, and DBL gradients, to attain concentration differences. Since microsensor measurements of O_2_, pH and Ca^2+^ were performed consecutively on different days, they did not depict true spatial replicates of one location (see also discussion ‘*Variability of microsensor measurements*’). Measurement position differences of ΔO_2_, ΔH^+^ and ΔCa^2+^ within individuals (Figure 1) were found to be non-significant. Consequently profiles (n = 2–4) were averaged for every individual for statistical analyses.

Means of ΔO_2_, ΔH^+^ and ΔCa^2+^ over replicate profiles per individual were tested for normality and homogeneity of variances by normality plots and Levene’s tests, respectively. Since parametric assumptions were violated, complete data sets of mean ΔO_2_, ΔH^+^ and ΔCa^2+^ were analyzed by Kruskal-Wallis one way analysis of variance, and alpha levels Bonferroni corrected (Table 2). Group comparisons were performed using Wilcoxon signed rank test (WSRT) for paired samples, Kruskal-Wallis one way analysis of variance and Wilcoxon rank sum tests ( = Mann Whitney U-tests) for unpaired samples, respectively. The ratios of mean ΔO_2_/ΔH^+^ of all individuals were compared across *pCO_2_* treatment groups using generalized linear models (GLMs). All statistical analysis used the software R [42] or SPSS 13.0 (IBM, Armonk, NY, USA).

**Table 2:** Omnibus Kruskal-Wallis one way analysis of variance results of mean (n = 2–4) ΔO_2_, ΔH^+^ and ΔCa^2+^ measured during three *pCO_2_* treatment incubations (432, 1141, 2151 µatm) for different factors.

	ΔO_2_ (µM)^a^			ΔH^+^ (nM)^a^			ΔCa^2+^ (µM)^a^		
	X^2^	df	p	X^2^	df	p	X^2^	df	p
*pCO_2_* treatment	0.0550	2	0.9729	14.8627	2	**0.0006**	2.1433	1	0.1432
illumination	44.175	1	**3.00e^−11^**	14.4391	1	**0.0001**	0.3827	1	0.5362
trophic level^b^	0.2462	1	0.6198	13.0502	1	**0.0003**	0.9686	1	0.3250
species	0.4675	5	0.9933	16.4631	5	**0.0056**	2.6097	5	0.7599
symbiont type^c^	0.3052	3	0.9591	15.6138	3	**0.0014**	2.5392	3	0.4682
treatment groups^d^	60.286	31	**0.0012**	58.0442	31	**0.0023**	12.556	23	0.9610

Significant effects at the Bonferroni corrected 0.83% levels are indicated in bold.

a Δ denotes the difference in O_2_, H^+^ and Ca^2+^ respectively between the surface of shell and the bulk seawater determined by microsensor profiling (n = 2–4), averaged over each individual.

b levels: photosymbiotic, heterotrophic.

c levels: diatoms (*Amphistegina radiata*, *Heterostegina depressa*), dinoflagellates (*Marginopora vertebralis*), red algae (*Peneroplis* sp.), no symbionts (*Quinqueloculina* sp., *Miliola* sp.).

d treatment groups represent each combination of species, *pCO_2_*, and light phase, according to box-plots represented in Figure 5, 6 and Figure S4.

## Results

### Individual Fitness

Both *Heterostegina depressa* at 2151 µatm and *Amphistegina radiata* individuals at 1141 and 2151 µatm showed visual signs of symbiont loss (i.e. bleaching) at the end of the 4 day incubations (Figure S2, S3). In *A. radiata*, bleaching was accompanied by severe symbiont clumping within the cell body.

### Zero-flow Experiment

**Figure 2:**
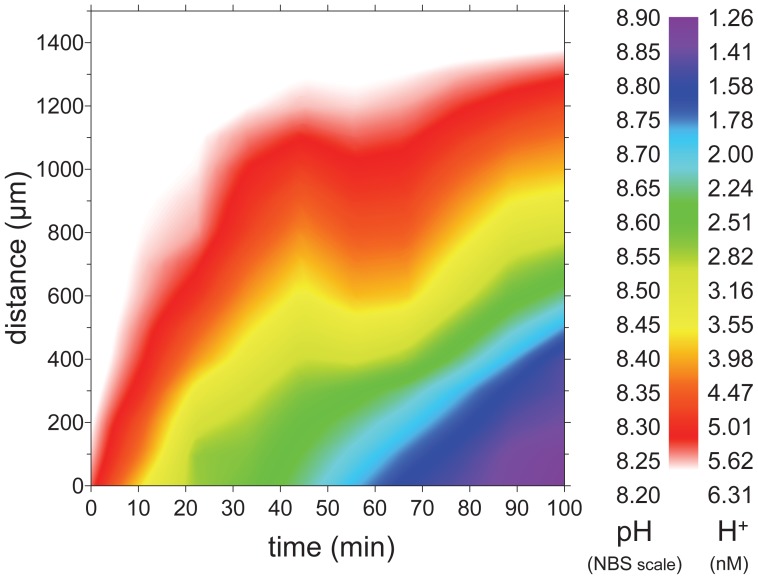
Temporal pH and H^+^ development of diffusive boundary layer (DBL) of *Marginopora vertebralis,* measured consecutively at a single position on the calcite shell at a *pCO_2_* of 432 µatm, 30 µmol photons m^−2^ s^−1^ under zero flow conditions after flow (0.5 cm s^−1^) was cut off at time = 0.

Within 30 sec after flow was turned off, no visible horizontal particle movement could be detected. Within 5 min after turning off the flow, pH gradients started increasing and after 100 min DBLs extended up to 1400 µm into the bulk seawater, reaching a maximum pH of 8.89 (1.29 nM of H^+^) at the surface of the shell (Figure 2). After flow was resumed, DBLs immediately reverted back to normal steady state conditions.

### O_2_ Microenvironment around Foraminiferal Tests

Due to their convex shapes, all foraminifera except for *M. vertebralis* had only few contact points with the bottom of the flow cell during the measurements. The effective thickness of the O_2_ DBL [21] on the tests (mean: 395±31 µm SE) ranged between 150 to 850 µm (Figure 1). In phototrophic specimens, DBL thickness was laterally enlarged where symbiont densities, and therefore photosynthetic activity, was higher than at the central part of the test. In *A. radiata, H. depressa* and *Peneroplis* sp., DBLs were also enlarged at the upstream edges. Differences of O_2_ between the shell surface and the bulk seawater, denoted as ΔO_2_, varied across the shell and among individuals, and were generally strongly elevated in photosymbiotic, and slightly reduced in symbiont-free species. The downstream edge of *M. vertebralis*, in which symbionts were sparse, exhibited a slight O_2_ under-saturation.

### Time Replicated O_2_ Dynamics within Individuals under Illumination

**Figure 3:**
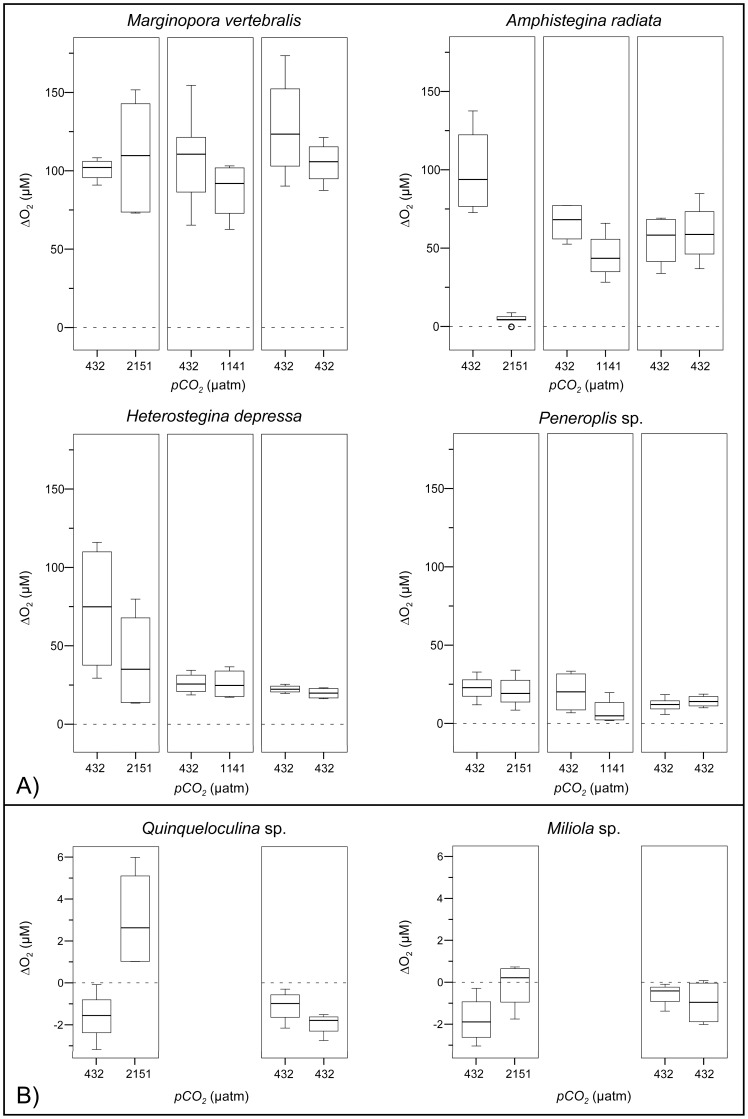
Box-plots representing the 25^th^, 50^th^ and 75^th^ percentiles of ΔO_2_, calculated from profiles measured within individuals (n = 2) prior (at 432 µatm) and during *pCO_2_* treatment incubations (432, 1141, 2151 µatm), under illumination (30 µmol photons m^−2^ s^−1^) for the six foraminiferal species. Note the different scales between A) photosymbiotic and B) symbiont-free species. Outliers (>1.5 interquartile range) are indicated by circles.

**Figure 4:**
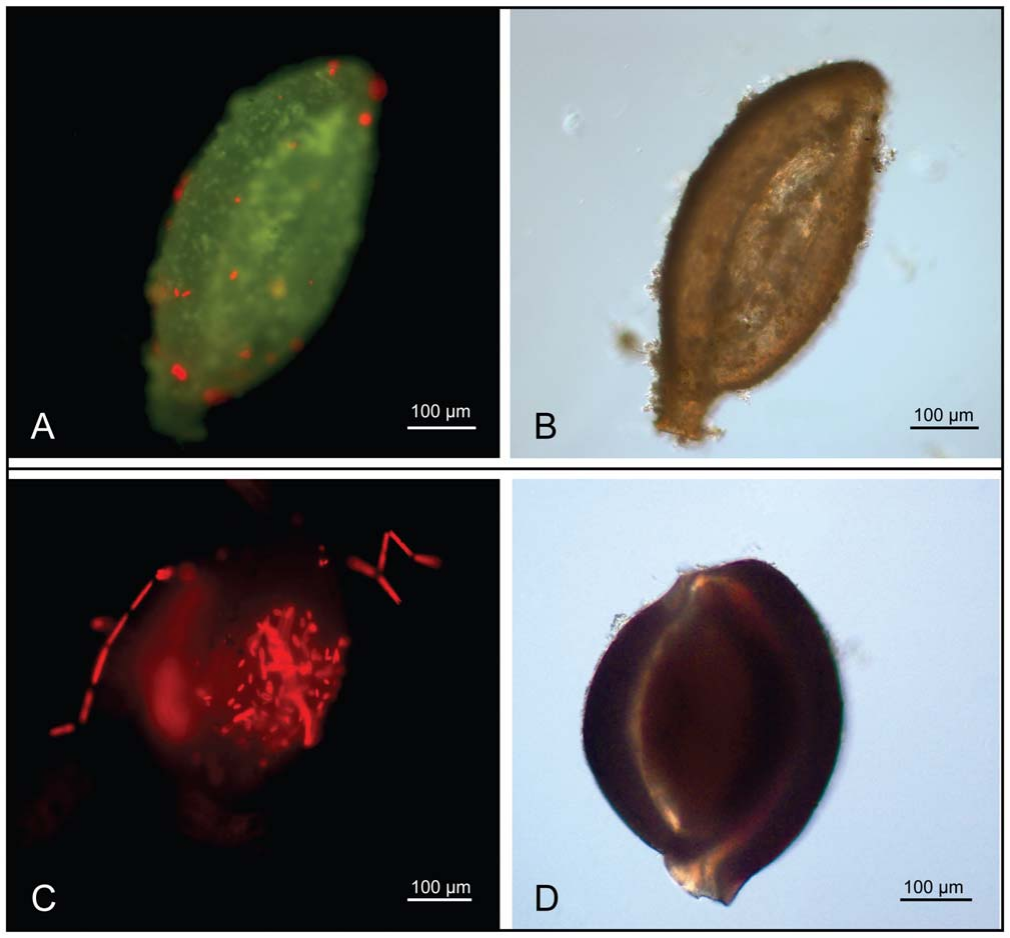
Exemplary microscopic images of *Quinqueloculina* (A, B) and *Miliola* (C, D) specimen profiled at 2151 µatm. (A, C) Chlorophyll autofluorescence (red) of phototrophic epiphytes on the calcite shell under green excitation light (FITC-filter set).

Within individuals, mean ΔO_2_ at 432 µatm (control measurements) remained constant, indicating the absence of confounding factors (WSRT, V = 33, p-value = 0.677, Figure 3). Only in *M. vertebralis* at 432 and 2151 µatm did ΔO_2_ variability increase from prior to during the incubations (Figure 3). This confirms that the repeated placement of microelectrodes on individuals did not affect readings. Variation in ΔO_2_ between individuals was greater for photosymbiotic than for symbiont-free species (Figure 3) and highest for *M. vertebralis* (all *pCO_2_* treatments), *A. radiata* (prior to the incubation) and *H. depressa* (prior to and during the incubation) at 2151 µatm. Under illumination mean ΔO_2_ was significantly elevated at all *pCO_2_* treatments in photosymbiotic, compared to symbiont free species (44±8 µM, vs. **−**0.002±0.753 µM (mean±SE), U-test: W = 0, p = 1.90e**^−^** ^07^). Beside individuals that exhibited symbiont loss, mean ΔO_2_ of photosymbiotic species did not change significantly between elevated and control *pCO_2_* (WSRT: V = 11, p = 0.106). In *A. radiata*, which displayed severe visual signs of bleaching, ΔO_2_ was strongly decreased at 2151 µatm (Figure S3). ΔO_2_ of symbiont-free individuals remained usually negative, very low and similar at both *pCO_2_* treatments. Yet, some profiles of positive ΔO_2_ (i.e. net photosynthesis) were measured in both *Quinqueloculina* (at 2151 µatm) and *Miliola* specimens (Figure 3). Subsequent fluorescence imaging revealed chlorophyll autofluorescence of epiphytes on the shell surfaces of these symbiont-free individuals (Figure 4).

### O_2_, H^+^ and Ca^2+^ Dynamics within and between Treatment Groups

**Figure 5:**
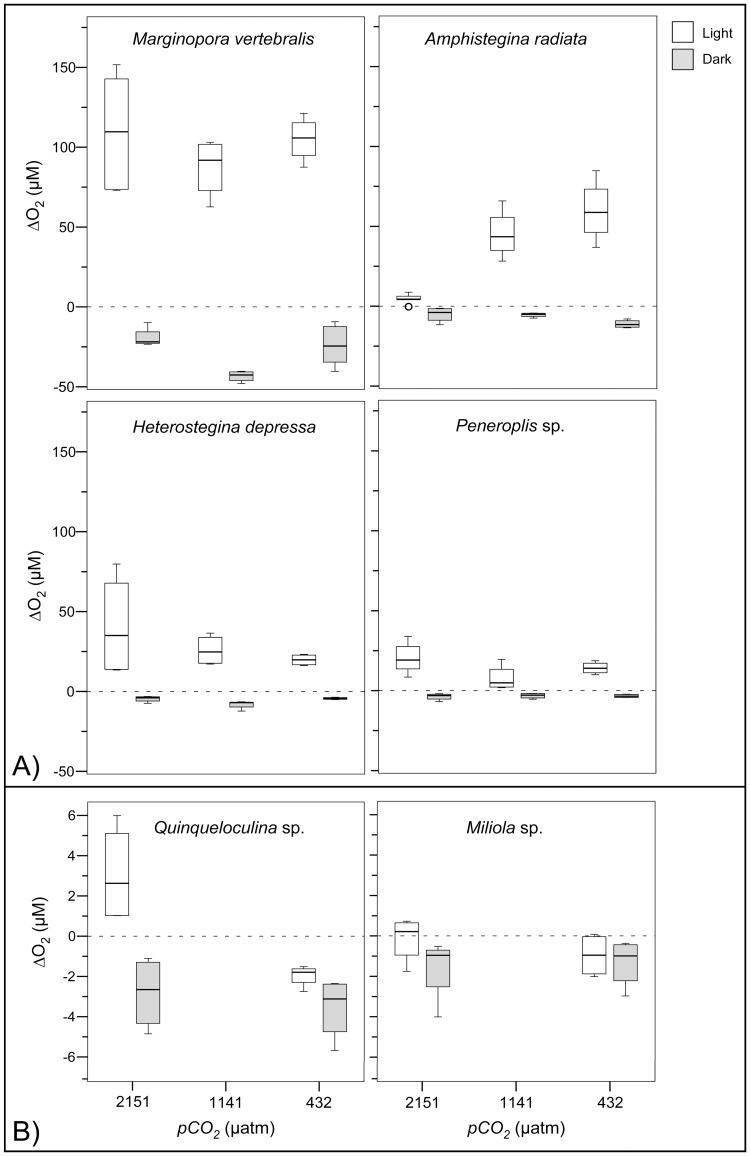
Box-plots representing the 25^th^, 50^th^ and 75^th^ percentiles of ΔO_2_, calculated from profiles measured during the *pCO_2_* treatment incubations, at light (30 µmol photons m^−2^ s^−1^) and dark conditions for the six foraminiferal species. Note the different scales between A) photosymbiotic and B) symbiont-free species. Outliers (>1.5 interquartile range) are indicated by circles.

**Figure 6:**
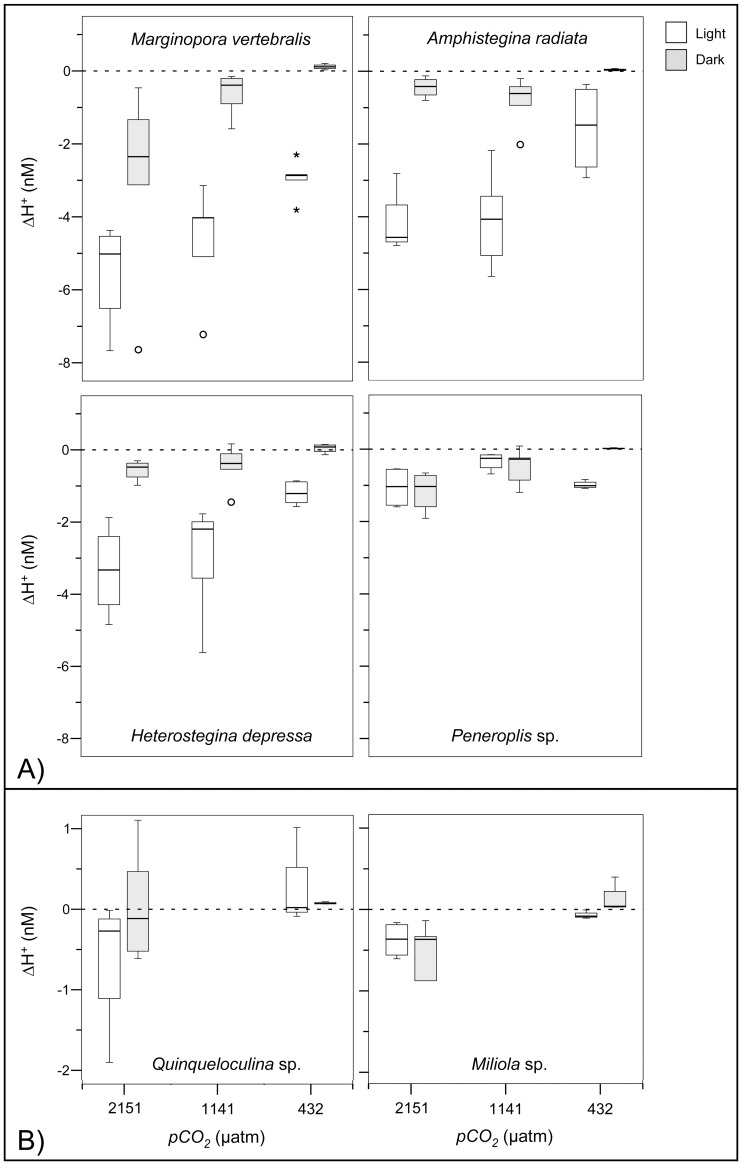
Box-plots representing the 25^th^, 50^th^ and 75^th^ percentiles of ΔH^+^, calculated from profiles measured during the *pCO_2_* treatment incubation, at light (30 µmol photons m^−2^ s^−1^) and dark conditions for individual species. Note the different scales between A) photosymbiotic and B) symbiont-free species. Outliers (>1.5 interquartile range) and extreme values (>3 times interquartile range) are indicated by (O) and (*) respectively.

Illumination significantly increased mean ΔO_2,_ and decreased mean ΔH^+^ in photosymbiotic, compared to symbiont-free species at all *pCO_2_* and between light and dark, indicating net photosynthesis (Table 2, Figure 5, 6). Beside *A. radiata* specimens, which strongly bleached at the highest *pCO_2_* level (Figure S3), mean ΔO_2_ in light did not change significantly between *pCO_2_* treatments (Kruskal Wallis: X^2^ = 1.8584, df = 2, p = 0.395). In darkness mean ΔO_2_ was negative in all photosymbiotic species indicating respiration (**−**11±3 µM), which was enhanced in *M. vertebralis* and *H. depressa* at 1141 µatm and reduced in *A. radiata* at increased *pCO_2_* (Figure 5). Symbiont-free species showed net respiration in both light and dark (**−**1.17±0.54 µM).

In contrast to ΔO_2_, mean ΔH^+^ was significantly affected by *pCO_2_* treatment, trophic level, species and symbiont-type (Table 2). Under illumination, mean ΔH^+^ of all photosymbiotic species decreased with increasing *pCO_2_* (**−**1.67±0.35 nM at 432 µatm vs. **−**3.53±0.66 nM at 2151 µatm, Figure 6), with the exception of *Peneroplis* individuals, where net photosynthesis was low and variable between the *pCO_2_* treatments (Figure 5). In darkness at 432 µatm, mean ΔH^+^ (0.070±0.019 nM) of all species was slightly increased indicating net respiration. Yet, all photosymbiotic species showed a negative mean ΔH^+^ at elevated *pCO_2_* conditions in darkness (**−**0.88±0.21 nM, Figure 6). ΔH^+^ of symbiont-free species was generally much lower in light (**−**0.20±0.15 nM), compared to photosymbiotic species and also slightly negative at 2151 µatm at both light levels (**−**0.49±0.17 nM).

Changes in mean ΔCa^2+^ were generally very low and exhibited high variation in space and time (39±24 µM). Mean ΔCa^2+^ did not change significantly with any of the measured factors (Table 2, Figure S4). At 2151 µatm mean ΔCa^2+^ was still not significantly different from 0 (23±29 µM), indicating no net CaCO_3_ dissolution or Ca^2+^ uptake.

### Ratios of Mean ΔO_2_/ΔH^+^ Across pCO_2_ Treatments

**Figure 7:**
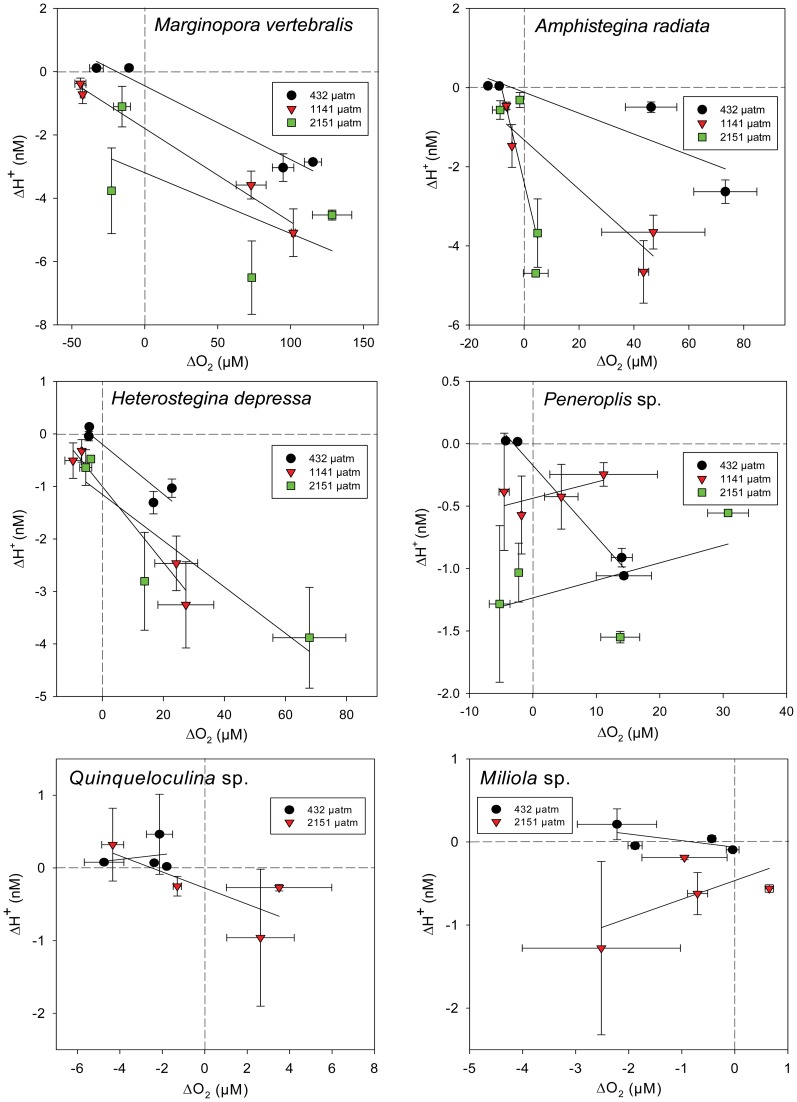
Relationship between mean ΔH^+^ and ΔO_2_ at different *pCO_2_* treatment groups for light (30 µmol photons m^−2^ s^−1^) and dark conditions. Each point represents an individual foraminiferal test (mean ± SE, n = 2–4). Solid lines indicate linear correlations for the different *pCO_2_* treatment groups, dashed lines indicate the respective ΔO_2_ and ΔH^+^ zero-lines.

Mean ΔO_2_ (i.e. netPS or respiration) and ΔH^+^ were both quite variable across profiles within and across individuals (Figure 7). Yet, there was a significant linear correlation between mean ΔO_2_ and mean ΔH^+^ (R^2^≥0.63, p_tm_<0.0166) per individual for all photosymbiotic species, but not in symbiont-free species (Table 3). The intercepts of the ΔO_2_/ΔH^+^ correlations were significantly decreased at increased *pCO_2_*, except in *H. depressa* (Figure 7, Table 3). In symbiont-free species, mean ΔO_2_ did not strongly correlate with ΔH^+^ (R^2^≤0.76, p_tm_>0.101), nor were intercepts and slopes of the regressions significantly different between the two *pCO_2_* treatments. Interestingly, ΔH^+^ of all linear regressions at ΔO_2_ = 0 was negative (range: **−**3.193 to **−**0.063 nM), beside *Quinqueloculina* at 432 µatm, indicating that H^+^ concentrations on the foraminiferal surface are slightly decreased compared with the bulk seawater when the net O_2_ flux equals zero.

**Table 3:** Relationships between ΔO_2_ and ΔH^+^ per individual within each species at different *pCO_2_* treatments (Figure 7).

	Estimate	SE	t	p	R^2^	p_tm_
***Marginopora vertebralis***						
Intercept	−36.005	12.244	−2.941	**0.0187**	0.80	**0.0036**
ΔO_2_	0.0116	0.1612	0.072	0.9446		
*pCO_2_* treatment	4.3394	1.5518	2.796	**0.0233**		
ΔO_2_ : *pCO_2_* treatment	−0.0045	0.0204	−0.222	0.8299		
***Amphistegina radiata***						
Intercept	−28.620	11.744	−2.437	**0.0408**	0.70	**0.0166**
ΔO_2_	−1.0420	0.5022	−2.075	0.0717		
*pCO_2_* treatment	3.4731	1.4950	2.323	**0.0487**		
ΔO_2_ : *pCO_2_* treatment	0.1237	0.0622	1.989	0.0819		
***Heterostegina depressa***						
Intercept	−14.865	6.4727	−2.297	0.0507	0.85	**0.0012**
ΔO_2_	0.1693	0.3218	0.526	0.6132		
*pCO_2_* treatment	1.7840	0.8203	2.175	0.0613		
ΔO_2_ : *pCO_2_* treatment	−0.0283	0.0417	−0.680	0.5159		
***Peneroplis*** ** sp.**						
Intercept	−13.038	3.7634	−3.464	**0.0085**	0.63	**0.0371**
ΔO_2_	0.8398	0.2972	2.826	**0.0223**		
*pCO_2_* treatment	1.5781	0.4768	3.310	**0.0107**		
ΔO_2_ : *pCO_2_* treatment	−0.1090	0.0382	−2.853	**0.0214**		
***Quinqueloculina*** ** sp.**						
Intercept	−6.7470	5.7909	−1.165	0.309	0.66	0.1885
ΔO_2_	−1.8837	1.9174	−0.982	0.382		
*pCO_2_* treatment	0.8514	0.7536	1.130	0.322		
ΔO_2_ : *pCO_2_* treatment	0.2333	0.2498	0.934	0.403		
***Miliola*** ** sp.**						
Intercept	−5.3733	4.0374	−1.331	0.254	0.76	0.1006
ΔO_2_	3.9626	2.7794	1.426	0.227		
*pCO_2_* treatment	0.6460	0.5145	1.256	0.278		
ΔO_2_ : *pCO_2_* treatment	−0.4918	0.3538	−1.390	0.237		

R^2^ constitutes the multiple R^2^ explaining total variance of the overall model.

P_tm_ depicts the significance of the total model. Linear-model results with significant effects at the 5% level are indicated in bold.

## Discussion

### ΔO_2_, ΔH^+^ and ΔCa^2+^ Dynamics

To test whether OA induced increases of seawater DIC enhance photosynthesis of photosymbiotic foraminifera and consequently result in increased pH levels within their microenvironments, we conducted microenvironmental O_2_ and pH measurements of photosymbiotic and symbiont-free foraminifera. In light, net O_2_ evolution (photosynthesis) within the DBL of photosymbiotic species remained relatively unaffected by the *pCO_2_* treatments and surface pH was significantly increased. Yet, H^+^ differences (ΔH^+^) were significantly enlarged within the DBL with increasing *pCO_2_*. However, the H^+^ decreases only amounted to ∼27% (at 432 µatm) and ∼14% (at 2151 µatm) of the ambient seawater H^+^ concentration. Photosynthesis was thus was insufficient to compensate for the more than four-fold increased ambient H^+^ concentrations between the highest and lowest *pCO_2_* treatment (Table 4). Rates of net photosynthesis of marine phototrophs primarily depend on temperature, nutrients and light availability, as well as the efficiency of the individual carbonate concentration mechanisms (CCMs, [43]–[45]). Except for bleached individuals, ΔO_2_ (i.e. net photosynthesis) was not influenced by *pCO_2_* in any species (Table 2, Figure 3). Since light levels were saturated and nutrient concentrations and temperature remained constant throughout each treatment, this may indicate either that the photosynthesis of photosymbiotic foraminifera was CO_2_ saturated at ambient *pCO_2_* concentrations, or that a down-regulation of DIC uptake occurred at increased *pCO_2_*. This notion is in agreement with previous studies on diatoms [46], [47] and *Symbiodinium* sp., both in culture and *in hospite* of corals [48], [49] and foraminifera [50], displaying a down-regulation of CCMs and only slight effects of increased DIC on net O_2_ evolution. Since there is no indication that the photosynthetic quotient (O_2_/CO_2,_ [51]) of the holobiont was altered at increased *pCO_2_*, DIC uptake should have been constant. Increases of *pCO_2_* on the other hand, cause a decrease in the CO_2_ uptake capacity of seawater (i.e. an increase of the Revelle factor, Table 1). This results in larger shifts of CO_2(aq)_, thus H^+^ concentrations in response to constant DIC production−/consumption-rates (for an extensive discussion of this aspect of carbon chemistry see [18], [52] and [53] Chapter 1.5). This will lead to stronger H^+^-gradients in response to constant photosynthesis/respiration rates at elevated *pCO_2_,* as indicated by the results (Figure 6; see also [18]). It is supported by the linear regression analyses, displaying a significant *pCO_2_* treatment effect on mean Δ*O_2_/*Δ*H^+^* of most photosymbiotic species (Table 3), and by previous modeling results of microenvironmental dynamics around phytoplankton, showing increased microenvironmental H^+^ variability at elevated *pCO_2_* [18].

**Table 4:** Ambient H^+^ concentrations and microenvironmental H^+^ differences (ΔH^+^) of photosymbiotic foraminifera at saturated light conditions (mean±SE).

*pCO_2_* treatment	ambient H^+^ (nM)	ΔH^+^(nM)
432 µatm	6.06±0.07	−1.67±0.35
1141 µatm	14.3±0.17	−2.92±0.63
2151 µatm	24.9±0.77	−3.53±0.66

The decreases of ΔO_2_, observed between 432 µatm and the elevated *pCO_2_* conditions in *A. radiata* at 1141 and 2151 µatm and *H. depressa* at 2151 µatm (Figure 3), are most likely the cause of increased symbiont loss (i.e. bleaching) at elevated *pCO_2_* (Figure S2, S3, [54], [55]). Additionally, bleaching and spatial variability of symbionts (see ‘variability of microsensor measurements’) in *A. radiata* and *H. depressa* resulted in severe symbiont clumping and increased heterogeneity of ΔO_2_ and ΔH^+^ across their shells. This might have led to an overestimation of the mean H^+^ difference (ΔH^+^) in light, in respect to the mean O_2_ difference (ΔO_2_), by profiling areas of high symbiont densities with pH sensor and areas of low symbiont density with O_2_ sensors (Figure 5, 6). This might explain why decreases of the ΔO_2/_ΔH^+^ intercepts in response to increased *pCO_2_* were less significant in *A. radiata* and slightly non significant in *H. depressa,* compared to all other photosymbiotic species (Figure 7, Table 3).

In dark, respiratory changes of ΔO_2_ and ΔH^+^ at 432 µatm were minor (Figure 5, 6). This is in agreement with previous microsensor measurements on foraminifera and diatoms [14], [15], [56], indicating that net respiratory O_2_ fluxes are generally very low in these protists.

Interestingly, microenvironmental H^+^ concentrations of all species were slightly decreased in darkness, compared to the bulk seawater at elevated *pCO_2_* (Figure 6). One possible reason for this may be the dissolution of the calcite shell at elevated *pCO_2_* in darkness, causing a local increase in pH [53]. However, this is unlikely, due to the absence of significant Ca^2+^ fluxes (Figure S4), and since Ω_Ca_ was super-saturated at even the highest *pCO_2_* (Table 1), indicating no net calcite dissolution. Another possibility could be the continued uptake of CO_2(aq)_ (>10 min) in the dark for CO_2_ fixation in the calvin cycle. This would however imply that CO_2(aq)_ uptake and fixation of the holobiont outweighed respiratory CO_2(aq)_ production in darkness. A third explanation could be that foraminifera actively up-regulate their microenvironmental pH in darkness, via active proton pumping or antiporter exchange [23], [57] into the cell, to compensate for increased seawater *pCO_2_* and to maintain pH homeostasis for vital cellular functions. A fourth explanation could be the excretion of nitrogen waste by the foraminifera in the dark in the form of NH_3_, which would elevate microenvironmental alkalinity, thus increase pH. The excretion of NH_3_ is widely distributed among marine protists [58]–[59] and might be increased at elevated *pCO_2_*, due to increased energy demands and nutrient uptake.

Mean ΔCa^2+^ over the shell surface was very low, but single profiles displayed strong gradients (Figure S4). Calcification in foraminifera, i.e. chamber formation, is discontinuous and sensitive to mechanical disturbances [23], [60], [61]. Due to the experimental handling it can be excluded that individuals were calcifying or preparing for chamber formation >2 h before and after the measurements. Increased Ca^2+^ uptake due to calcification was therefore not expected. The measured high variability and averaged low fluxes of ΔCa^2+^ over the shell surface are in accordance with previous microsensor measurements on tropical (*Marginopora vertebralis*, *Amphistegina lobifera*, [15]) and temperate benthic (*Ammonia* sp., [23]) and planktonic (*Orbulina universa*, [16]) specimens. This indicates that Ca^2+^ exchange varies over time and is not evenly distributed over the shell surface for most foraminifera, but very localized. As Ca^2+^ is an important cellular ionic regulator and cytotoxic at increased cellular concentrations [62], its exchange via Ca^2+^ channels in the protoplasmic membrane must be highly regulated. Distribution of Ca^2+^ channels and Ca^2+^ fluxes over the foraminiferal surface are most likely patchy. Ca^2+^ gradients would therefore only affect a small percentage of the total foraminiferal surface area, which would lead to the generally low total Ca^2+^ fluxes, but high variability in different profiles as observed (Figure S4).

### Characterizing the Foraminiferal Microenvironment

O_2_ and pH DBL dynamics of photosymbiotic foraminifera and other photosynthetic calcifiers correlate in response to illumination changes, with pH dynamics exhibiting a temporal time lag following O_2_ dynamics [13], [16], [17], [19].

The extent to which surface O_2_ and pH on the organisms’ surface deviate from the bulk seawater depends on multiple factors, such as the photosynthetic activity of the organism, surrounding seawater flow, seawater H^+^-buffering capacity, diffusivity/permeability of CO_2_ from its source – spatial configuration of symbiont and host, diffusional transport constrains (1–3D) and the 3D surface structure of the location [15], [17], [21], [53], [63]. Since carbonate chemistry remained constant throughout the treatments (Table 1), most prominent factors during the experiment influencing DBL dynamics, included diffusional transport constrains to and from the symbionts, micro-flow surface dynamics and location specific rates of net photosynthesis and respiration. This is illustrated by the spatial extent of the DBLs (Figure 1, 2). The thickness of the ΔO_2_ DBL clearly decreases along middle ridges of individuals, where laminar flow velocity was highest [64], [65] and underlying photosynthesis was lower, due to decreased symbiont density in that region, compared to lateral symbiont-rich parts (Figure 1, Figure S1). *M. vertebralis* specimens showed the steepest O_2_ and pH gradients, without enlarged DBL thickness (i.e. net O_2_ fluxes), indicating overall increased photosynthesis compared to all other species (Figure 1, 3, 5). Yet, ventral sides of *M. vertebralis* specimens locked tightly on to the inert surface of the flow chamber, thereby creating a one-dimensional diffusional barrier. The strong O_2_ and pH microgradients of *M. vertebralis* can therefore not solely be attributed to increased photosynthesis but emerge as a combination of the underlying photosynthesis, flat surface structure (and thereby almost parallel horizontal emerging flow field), as well as one-dimensional diffusional resistance.

### Variability of Microsensor Measurements

Measurement variability was high, but much higher between, than within individuals (Figure 3, 5, 6, 7), allowing for temporal replication of microsensor measurements. Variability was not unexpected due to the typically high spatial variability of O_2_ fluxes and pH dynamics across the surface of photosynthetic organisms (Figure 1, [15], [19]–[21], [65]) in combination with the high spatial resolution of the microsensor measurements (reviewed in [28]). Another source of variability is due to the fact that some foraminiferal species, including *M. vertebralis* and *H. depressa*, actively transport their symbionts within their cell bodies and individual chamberlets [66], [67], resulting in higher variation of ΔO_2_ (Figure 3) and consequently ΔH^+^ over time for a specific spot on their shell surface. Spatial variability of ΔO_2_ and consequently ΔH^+^ (and their means), measured within and among the individuals, was therefore expected. Yet, spatial heterogeneity within individuals (Figure 1) was not represented in the sampling, since measurement positions were not significantly different. Also ΔO_2_, measured before and during the 432 µatm treatment under equal conditions (Figure 3) within the same individuals, remained relatively constant, confirming that the spatial placement of microelectrode measurements could be replicated.

### Mixed Responses of Ocean Acidification Experiments

Several studies have reported contrary responses of increased *pCO_2_* on both photosynthesis and calcification on a variety of marine taxa [11], [12], [68]–[70], even within phyla (reviewed in Doney et al. [1]). Possible causes for such variability are diverse, potentially including differences in calcifying-/carbonate concentration mechanisms and their coupling, tolerance levels, adaptation mechanisms, but also differences in the experimental designs and setups. Consequently, a comparison among ocean acidification studies, even within phyla, is difficult. Especially flow, as an important experimental parameter influencing the surface pH of organisms, has not been considered in many ocean acidification experiments. Yet, flow changes are well known to severely impact microenvironmental pH levels of photosymbiotic foraminifera (Figure 2, [15]) and other phototrophs in light [19], [20], [63]. The changes in surface pH are especially severe within static culture experiments, where ΔpH can change up to >1 unit (>5 nM of H^+^, Figure 2, [15], [71]). Zero-flow conditions for ocean acidification studies should therefore be avoided, as they are ecologically unrealistic and also confuse the carbonate chemistry of the intended *pCO_2_* perturbation, causing unrealistically high/low microenvironmental pH conditions in light/dark, despite increased DIC levels.

### Effects of Ocean Acidification on Benthic Foraminifera

It appears that benthic foraminifera do not show uniform responses to low pH conditions [50], [70], [72]–[82]. While most laboratory studies investigating calcification in symbiont-free foraminifera showed decreases in calcification rate [72], [74], [75], [82] larger photosymbiotic foraminifera show more variable responses [50], [70], [76], [77]. Also, experiments conducted under low and stagnant flow conditions exhibit mostly decreases in calcification rate [70], [72]–[75], [82], while experiments applying higher rates of turbulent mixing show variable responses on rates of calcification, net photosynthesis and respiration ([50], [76], [77], this study). Thus calcification responses seem to correlate to some extend with the experimental flow conditions as suggested for corals (reviewed in [83]). While for symbiont-free shallow infaunal/epibenthic foraminifera low flow conditions (<0.1 cm s**^−^** ^1^) represent ecological realistic values, mimicking pore-water flow and sediment surface friction velocities [23], [74], [75], [82] this is not the case for most epibiotic photosymbiotic species (also discussed in [50]). However, since experimental conditions are quite variable among the different studies, e.g. acid base manipulations, thus TA manipulations [72]–[74], versus *pCO_2_*, thus DIC manipulations ([50], [70], [75]–[77], [82], this study), a direct correlation between experimental flow conditions and calcification responses remains speculative. Yet, the here presented results strongly indicate that especially for larger benthic photosymbiotic foraminifera, the interplay between flow and net photosynthesis has severe impacts on microenvironmental pH, thus microenvironmental DIC availability.

Some of the observed variability in calcification responses of photosymbiotic foraminifera to OA are likely due to differences in calcification mechanism (also discussed in [84]–[86]), as well as solubility differences of the calcite tests [87], [88] of the different groups. This is represented in the literature showing unaffected or increased calcification rates in hyaline (low Mg-calcite: less soluble) and decreased rates in miliolid (high Mg-calcite: more soluble) species in response to elevated *pCO_2_* [70], [73], [76], [77]. These taxa specific differences are in line with previous studies on foraminiferal DIC uptake mechanism, showing almost linear increases in miliolid *Amphisorus hemprichii* and almost no change in *Amphistegina lobifera* in response to increasing DIC (and CO_3_^2−^) concentrations in the OA range between 2 and 3 mM ([84], also discussed in [76]). Additionally, these ideas are supported by recent field studies investigating foraminiferal assemblages at volcanic CO_2_ vents in the Mediterranean [79], [81] and in tropical coral reefs [80]. The studies in the Mediterranean reported significantly reduced numbers of calcareous species, a complete absence of miliolid and only the presence of hyaline species at elevated *pCO_2_* [79], [81]. The study investigating cold CO_2_ seeps within tropical coral reefs reported almost complete absence of the larger epibiotic miliolid species *Marginopora vertebralis* and reduced species richness and diversity of sedimentary foraminifera at high *pCO_2_* sites [80]. A very recent study investigated symbiont-free hyaline foraminiferal assemblages in a CO_2_ enriched, benthic habitat in the southwestern Baltic Sea [78]. This study showed that mainly sediment Ω_Ca_ under-saturation, rather than the *pCO_2_* levels of the sediments, determines the population density of the benthic shallow infaunal species *Ammonia ammoriensis*, yet not of *Elphidium incertum* [78]. These findings support the idea of increased resistance/adaptation of hyaline species within their natural habitat to high *pCO_2_* conditions, compared to miliolid species.

The findings of this study indicate that photosynthesis can only to a minor extend compensate for ambient seawater pH decreases within the microenvironment of photosymbiotic foraminifera (Table 4). Symbiont-free and photosymbiotic foraminifera are thus likely to experience strongly decreased microenvironmental pH conditions at future *pCO_2_*, making their cell bodies susceptible to the physiological effects of ocean acidification.

## Supporting Information

Figure S1 **Close up of the six foraminiferal species, photographed via dissecting microscope (A–E) and back-light microscope (F).** Images were taken after control (432 µatm) treatment incubations. A) *Marginopora vertebralis*, B) *Amphistegina radiata*, C) *Heterostegina depressa*, D) *Peneroplis* sp., E) *Quinqueloculina* sp. and F) *Miliola* sp. individuals. Sizes are stated as largest possible diameter of individuals.
(TIF)

Figure S2 **Close up dissecting microscope images, taken before (432 µatm) and after the 1141 µatm treatment incubation.** A) *Marginopora vertebralis*, B) *Amphistegina radiata*, C) *Heterostegina depressa*, D) *Peneroplis* sp., individuals. Sizes are given as largest possible diameter of individuals. In *A. radiata,* areas of bleaching are indicated by white dashed circles.
(TIF)

Figure S3 **Close up dissecting microscope images, taken before (432 µatm) and after the 2151 µatm treatment incubation.** A) *Marginopora vertebralis*, B) *Amphistegina radiata*, C) *Heterostegina depressa*, D) *Peneroplis* sp., individuals. Sizes are stated as largest possible diameter of individuals. Both *A. radiata* and *H. depressa* showed signs of bleaching. Symbiont clumping in *A. radiata* is indicated by white dashed circles.
(TIF)

Figure S4 **Box-plots representing the 25^th^, 50^th^ and 75^th^ percentiles of ΔCa^2+^, calculated from profiles measured during the** ***pCO_2_*** **treatment incubation, at light (30 µmol photons m^−2^ s^−1^) and dark conditions for individual species.** Note the different scales between A) photosymbiotic and B) symbiont-free species. Outliers (>1.5 interquartile range) and extreme values (>3 times interquartile range) are indicated by (O) and (*) respectively.
(TIF)
