# Supplementary figures and images for: The O2, pH and Ca2+ Microenvironment of Benthic Foraminifera in a High CO2 World
Source: PLoS One. 2012 Nov 15;7(11):e50010. doi: 10.1371/journal.pone.0050010 (PMC3499438; doi:10.1371/journal.pone.0050010)

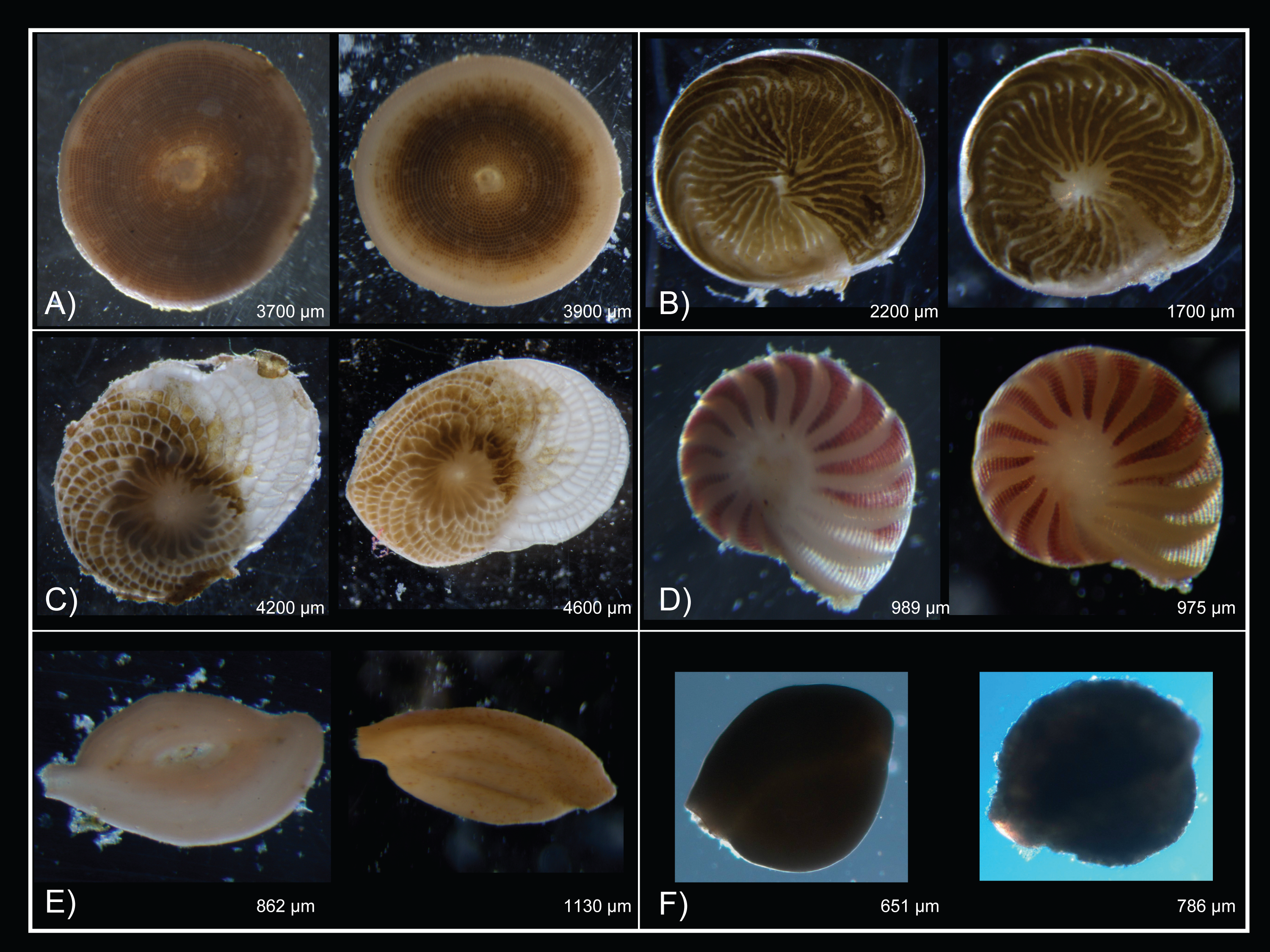

Supplement: Figure S1 — Close up of the six foraminiferal species, photographed via dissecting microscope (A–E) and back-light microscope (F). Images were taken after control (432 µatm) treatment incubations. A) Marginopora vertebralis, B) Amphistegina radiata, C) Heterostegina depressa, D) Peneroplis sp., E) Quinqueloculina sp. and F) Miliola sp. individuals. Sizes are stated as largest possible diameter of individuals. (TIF) [file pone.0050010.s001.tif]

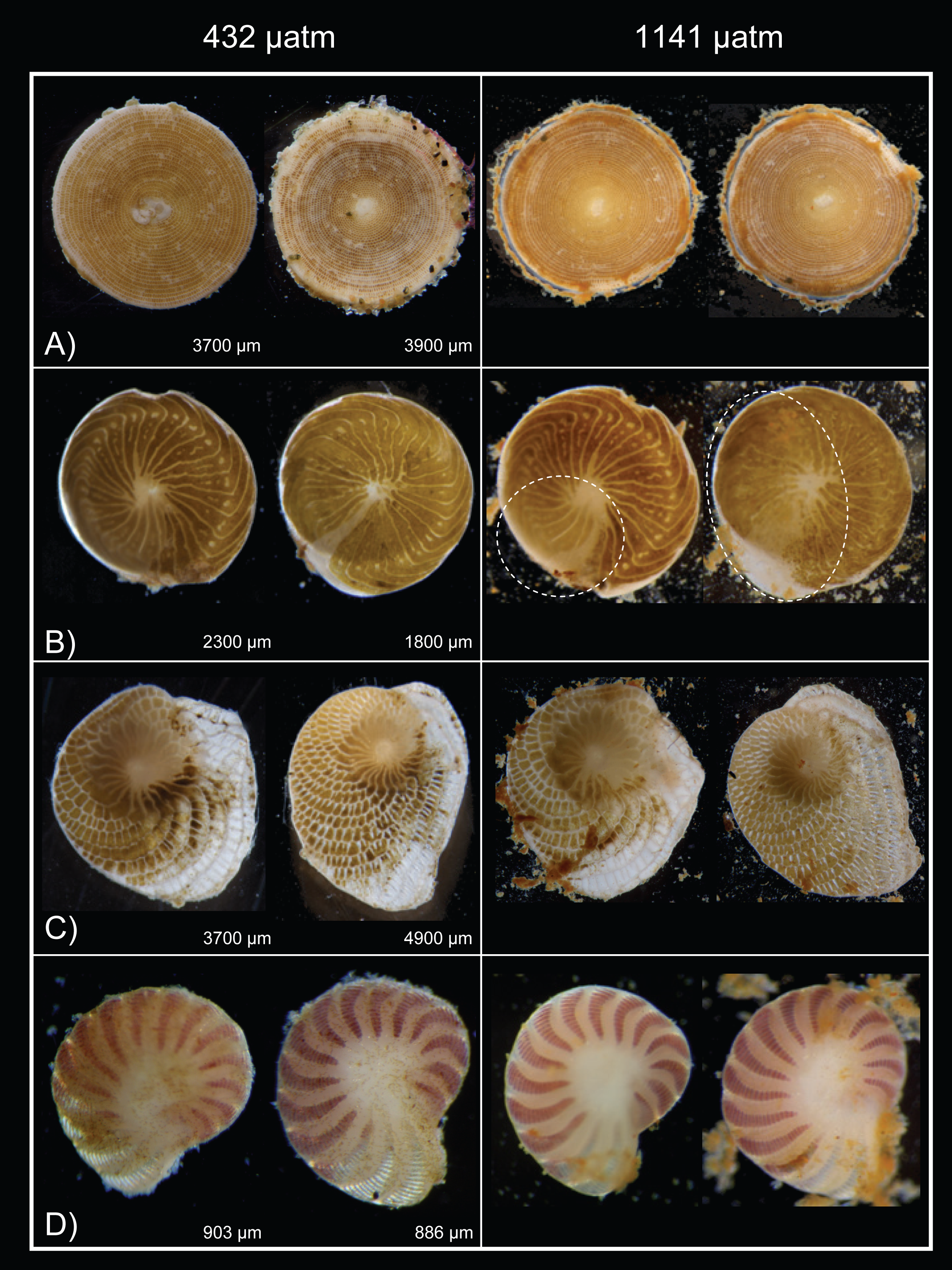

Supplement: Figure S2 — Close up dissecting microscope images, taken before (432 µatm) and after the 1141 µatm treatment incubation. A) Marginopora vertebralis, B) Amphistegina radiata, C) Heterostegina depressa, D) Peneroplis sp., individuals. Sizes are given as largest possible diameter of individuals. In A. radiata, areas of bleaching are indicated by white dashed circles. (TIF) [file pone.0050010.s002.tif]

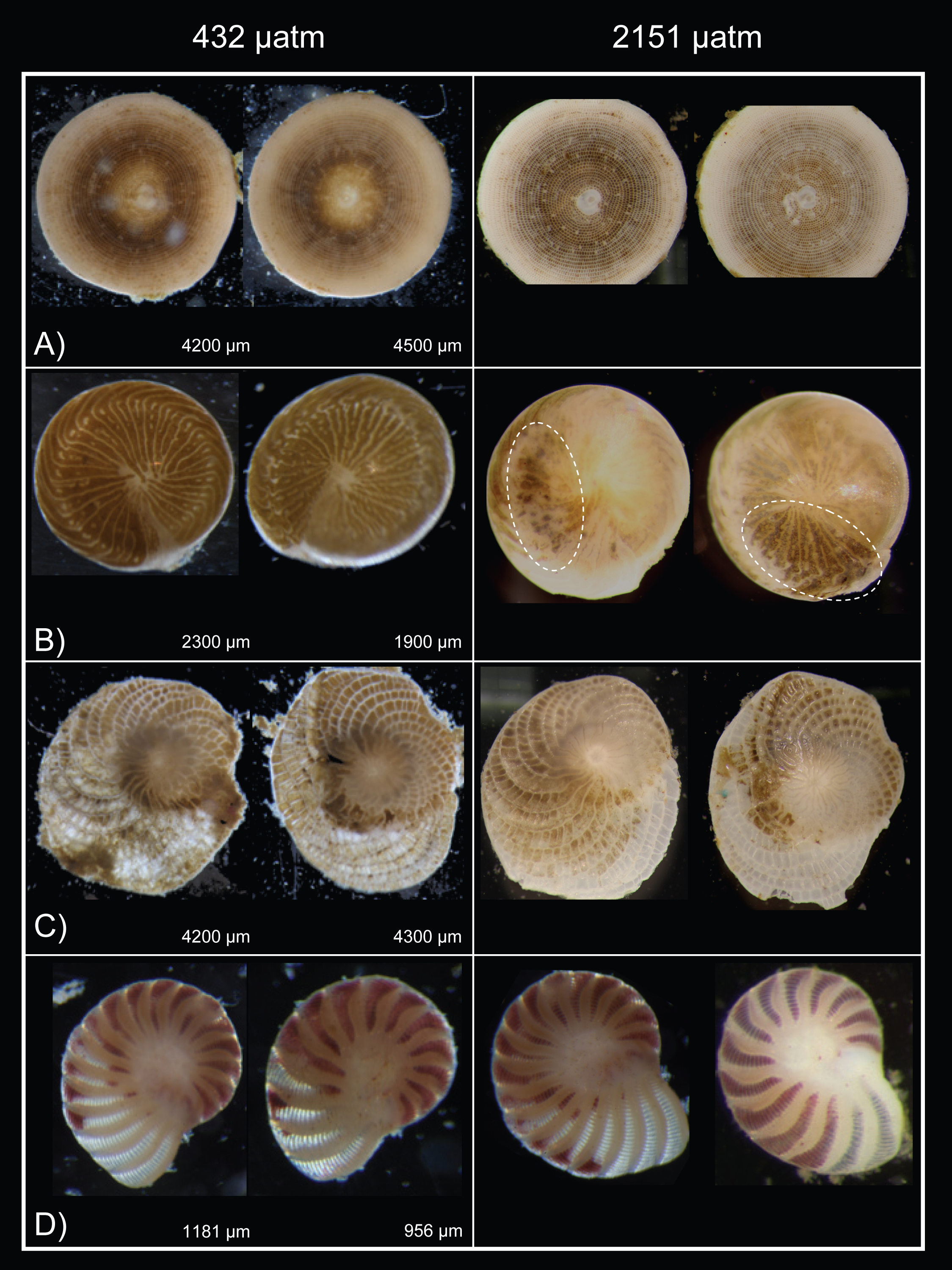

Supplement: Figure S3 — Close up dissecting microscope images, taken before (432 µatm) and after the 2151 µatm treatment incubation. A) Marginopora vertebralis, B) Amphistegina radiata, C) Heterostegina depressa, D) Peneroplis sp., individuals. Sizes are stated as largest possible diameter of individuals. Both A. radiata and H. depressa showed signs of bleaching. Symbiont clumping in A. radiata is indicated by white dashed circles. (TIF) [file pone.0050010.s003.tif]

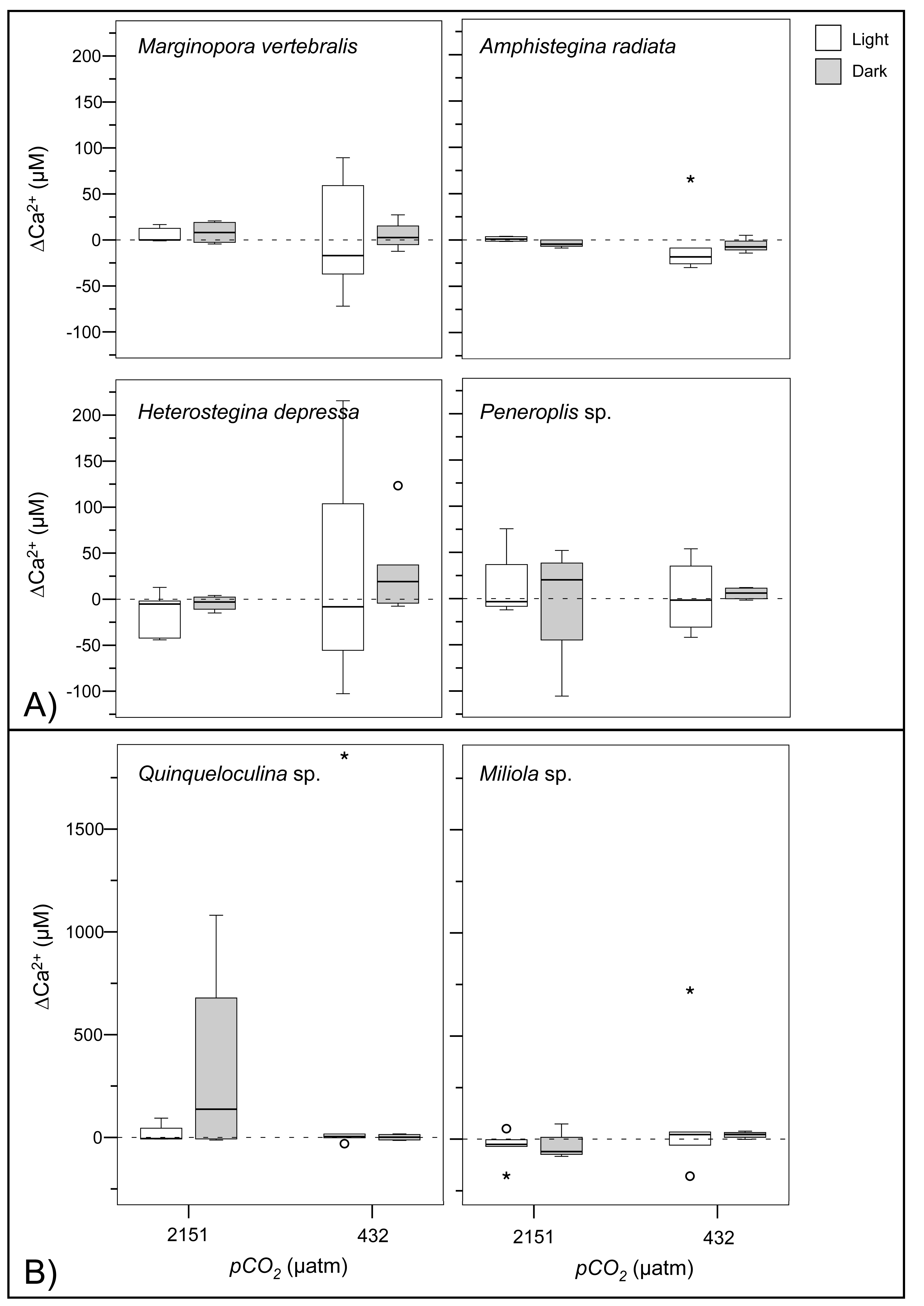

Supplement: Figure S4 — Box-plots representing the 25th, 50th and 75th percentiles of ΔCa2+, calculated from profiles measured during the pCO2 treatment incubation, at light (30 µmol photons m−2 s−1) and dark conditions for individual species. Note the different scales between A) photosymbiotic and B) symbiont-free species. Outliers (>1.5 interquartile range) and extreme values (>3 times interquartile range) are indicated by (O) and (*) respectively. (TIF) [file pone.0050010.s004.tif]
